# LC-MS determination of *Nicotiana benthamiana* host plant proteins in the drug products of recombinant plant-produced pembrolizumab

**DOI:** 10.1038/s41598-025-11541-6

**Published:** 2025-07-15

**Authors:** Ratama Daduang, Pipob Suwanchaikasem, Kaewta Rattanapisit, Sarocha Vitayathikornnasak, Theerakarn Srisangsung, Christine Joy I. Bulaon, Waranyoo Phoolcharoen

**Affiliations:** 1 Baiya Phytopharm Co., Ltd, Bangkok, 10330 Thailand; 2 Department of Pharmacognosy and Pharmaceutical Botany, Faculty of Pharmaceutical Sciences, Chulalongkorn University, Bangkok, 10330 Thailand; 3 Center of Excellence in Plant-produced Pharmaceuticals, Chulalongkorn University, Bangkok, 10330 Thailand

**Keywords:** Luminal-binding protein 5, RuBisCO, Fast protein liquid chromatography, Monoclonal antibody, Plant molecular farming, Protein purification, Biochemistry, Biotechnology, Plant sciences

## Abstract

Host proteins from *Nicotiana benthamiana* can remain in the recombinant biologic products after undergoing multiple steps of purification. These plant protein impurities may induce immunogenicity upon use. Therefore, controlling and monitoring host cell proteins is necessary throughout the process of recombinant protein production. Liquid chromatography-mass spectrometry (LC-MS) has been successfully used to characterize protein species down to nanogram levels in various types of samples. In this study, LC-MS was applied to detect *N. benthamiana* plant proteins in plant-produced pembrolizumab anti-cancer antibody. Two types of purification techniques, gravity flow with manually packed column and automated system with prepacked column, were studied. After purification, the protein products were primarily assessed with SDS-PAGE and Western blot analyses and further examined with LC-MS to confirm protein identity and investigate plant protein contaminations. The pembrolizumab sequence was confirmed with more than 89% coverage. A higher number of host plant proteins were detected in the protein samples purified with gravity flow column. Luminal-binding protein 5 and ribulose bisphosphate carboxylase (RuBisCO) enzyme were predominant host plant proteins detected. Luminal-binding protein 5 was observed in the products purified with both purification methods. It was likely bound to pembrolizumab antibody and co-eluted into eluate fraction as its sequence is similar to binding immunoglobulin protein (BiP). RuBisCO enzyme was detected in the samples purified with gravity flow only. Its presence was likely due to incomplete wash by gravity flow chromatography. In summary, this study provided an important clue for plant proteins that could be contaminated in plant-produced products and suggested that second column chromatography is required to enhance purification efficiency.

**Supplementary Information:**

The online version contains supplementary material available at 10.1038/s41598-025-11541-6.

## Introduction

Plant molecular farming has become an alternative source for recombinant protein productions, showing success in the productions of human vaccines, antibodies, serum albumin, avidin and β-glucocerebrosidase^1,2^. When compared to other expression hosts, such as bacteria, yeast, insect and mammalian cells, plants offer a range of benefits, including absence of human pathogens and avoidance of animal uses, rolling public acceptance to plant-produced proteins^3^. Additionally, biowastes generated from plant molecular farming system are mostly biodegradable, promoting sustainability in plant-based factory^4^.

Transient expression system using *Nicotiana benthamiana* as a plant host and agroinfiltration as a method for gene delivery is a widely used platform in plant molecular farming^5^. Gene of target protein is incorporated into a desired plasmid and delivered to *N. benthamiana* plant using pathogenic bacteria, *Agrobacterium tumefaciens*, as a carrier^6^. After a few days of infection, plants are collected and extracted for crude protein, which is a mix of plant proteins and a target protein. Protein purification is then implemented to isolate target protein from host plant proteins. This step is critical and may require a combination of techniques, including flocculation, filtration, ion-exchange chromatography, affinity chromatography and inverse transition cycling, to promote purification efficiency^3,5^. Improper protein purification would confer contaminations from host cell proteins, downgrading product safety and efficacy^7^. Based on U.S. Pharmacopoeia (U.S.P.), residual host cell proteins in biopharmaceutical products should be less than 100 ng per mg protein^8^. Nonetheless, the standard is drawn based on the uses of *Escherichia coli* and mammalian cells as expression hosts. Host plant protein impurities have not been duly characterized, and the purification protocols to isolate recombinant protein purification from plant cells have not been systematically optimized^8^, requiring in-depth research to establish a good practice for purification and quality control of host cell proteins within plant molecular farming system.

In general, sandwiched enzyme-linked immunosorbent assay (ELISA) is a recommended protocol for host cell protein detection in cell-based systems^8^. However, it is unable to detect host cell proteins with weak immunoreactivity or low binding affinity against antibody epitopes^9^. An alternative technique is a liquid chromatography-mass spectrometry (LC-MS), a highly sensitive and robust technique for protein identification, characterization and quantification^10^. It is widely used in protein and biomarker discovery to diagnose and track down various diseases^11,12^. Therefore, LC-MS technique can be adapted for detecting host plant proteins in plant-produced protein products^13,14^.

At Baiya Phytopharm Co., Ltd., recombinant proteins, for example COVID-19 vaccines, anti-cancer monoclonal antibodies and plant-based growth factors, are produced using *N. benthamiana* transient expression system^15,16^. Anti-cancer pembrolizumab antibody is one of the leading products in our drug development scheme since it is highly expressed in *N. benthamiana* plants, providing good production yield^17^. However, protein purification and concentration are current issues of pembrolizumab production, requiring further optimization to obtain high-purity, concentrated proteins^17^. Generally, monoclonal antibody with human IgG based structure can be purified using protein A affinity column^18^. Nonetheless, protein purity cannot be guaranteed within a single step of purification, where host plant proteins might be co-purified with target protein.

**Fig. 1:**
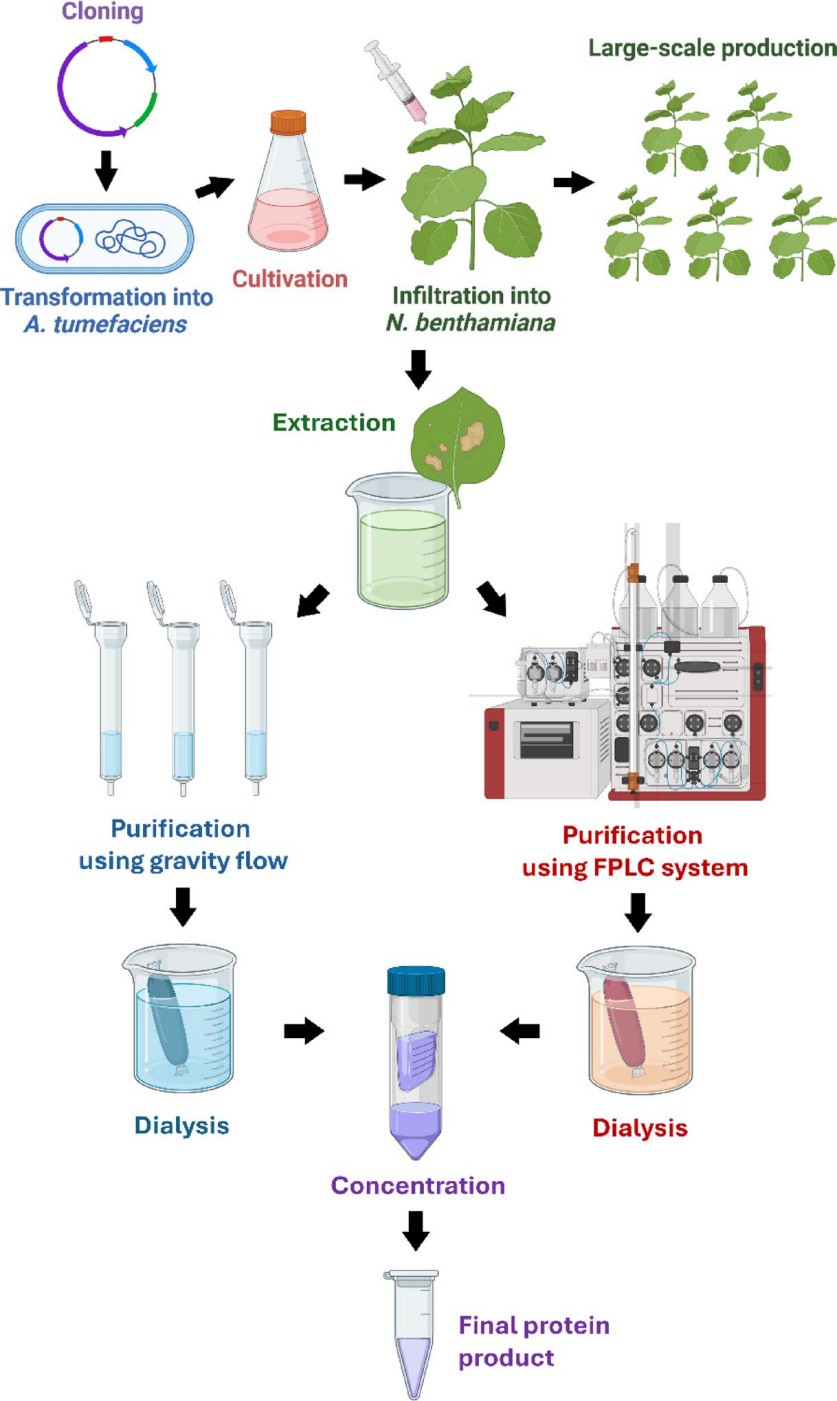
Workflow of recombinant pembrolizumab production in this study. The upstream processes were gene cloning, *A. tumefaciens* transformation, agroinfiltration and protein expression in *N. benthamiana* plants. The downstream process started when the crude extracts from 20 g of plant material for gravity flow purification method and the crude extracts from 50 g of plant material for pressure flow columns were loaded. Protein eluate was dialyzed and concentrated before subjected to SDS-PAGE, Western blot and LC-MS analyses. This figure was partly created with Biorender.com.

Therefore, this study aimed to apply LC-MS technique to characterize host plant proteins that could remain in the plant-produced pembrolizumab after purification with protein A affinity column. Two types of purification were studied for method comparison. The products purified with small-scale purification system using gravity flow for a manually packed column were compared with the products purified with pilot-scale purification system using fast protein liquid chromatography (FPLC) machine with protein A prepacked column (Fig. 1). All pembrolizumab samples were adjusted to 1 mg ml^−1^ before processed to LC-MS analysis. Host plant proteins detected were identified against *Nicotiana* database, and their abundances were compared with pembrolizumab proteins. The results provided an important clue to understanding the contamination of host plant proteins upon the use of protein A chromatography and could be used to improve purification efficiency in the production of pembrolizumab antibody under plant molecular farming system.

## Results

### Characterization of pembrolizumab proteins

**Fig. 2:**
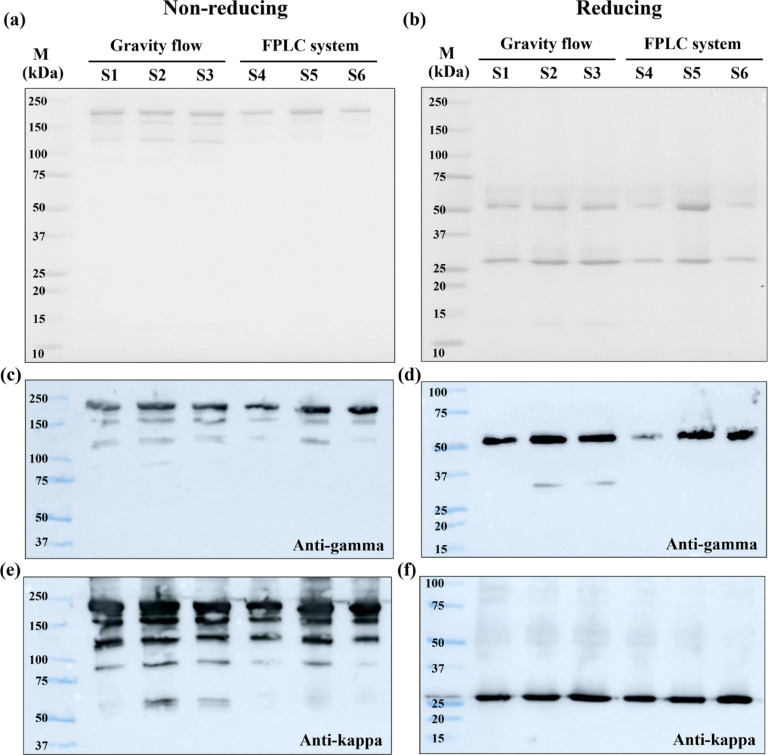
SDS-PAGE and Western blot analysis of six pembrolizumab drug products. The first three samples (S1–S3) were purified with manually packed protein A column. The last three samples (S4–S6) were purified with prepacked protein A column equipped with FPLC system. Stained with Coomassie Blue, SDS-PAGE gels under non-reducing and reducing conditions are shown in (**a**) and (**b**), respectively. Western blot results of non-reducing and reducing gels, probed with anti-human IgG gamma antibody, are shown in (**c**) and (**d**), respectively. Western blot results of non-reducing and reducing gels, probed with anti-human IgG kappa antibody, are shown in (**e**) and (**f**), respectively. M is a Precision Plus protein marker (Bio-Rad, US).

After completing protein purification processes, the final pembrolizumab products were primarily assessed using SDS-PAGE and Western blot analyses. The yields of pembrolizumab obtained from gravity flow and pressure flow purification system were 10.60 ± 2.82 and 6.85 ± 4.73 mg per 1 kg plant, respectively. Expected bands of pembrolizumab were observed in all six protein samples. In non-reducing conditions, the most intense protein band at approximately 200 kDa was deemed as intact pembrolizumab protein (Fig. 2a). The smaller bands between 150 and 100 kDa could be degraded forms of pembrolizumab. These bands were more obvious in the protein products purified with gravity flow (sample 1–3) as compared to the proteins purified with pressure flow (sample 4–6). In reducing conditions, the band of pembrolizumab heavy chain was observed at approximately 50 kDa and the band of pembrolizumab light chain was seen at approximately 28 kDa in all six samples (Fig. 2b).

In Western blot analysis, pembrolizumab proteins were confirmed with anti-human gamma heavy chain and anti-human kappa light chain antibodies (Fig. 2c-f). Under non-reducing conditions, the bands at approximately 200 kDa of all six pembrolizumab samples were detected after probing with both anti-gamma (Fig. 2c) and anti-kappa antibodies (Fig. 2e). A few smaller bands were detected in the analysis, probed with anti-kappa antibody. These bands could be fragmented or truncated forms of pembrolizumab, that still contained the epitope which the anti-kappa antibody can bind to. Under reducing conditions, the band at approximately 50 kDa was detected with anti-gamma antibody (Fig. 2d) and the band at approximately 28 kDa was visualized with anti-kappa antibody (Fig. 2f), confirming that those bands were heavy and light chain of pembrolizumab, respectively. These findings demonstrated a successful isolation of recombinant pembrolizumab by the uses of both manual and systemic flow of protein A affinity column. Raw figures of SDS-PAGE and Western blot analyses were demonstrated in Supplementary Figure S1.

### Host plant protein identification in pembrolizumab drug products

In total, 20 protein groups were identified among all six pembrolizumab samples (Table 1). Two out of 20 proteins were target pembrolizumab heavy and light chains. They were the most abundant proteins, identified with 60 and 34 razor/unique peptides, respectively. The other 18 proteins were identified as *Nicotiana* plant proteins. Six of them, including luminal-binding protein 5, ribulose biphosphate carboxylase (RuBisCO) large chain, cysteine-rich venom protein, luminal-binding protein 5-like, RuBisCO small subunit and plastocyanin A’/A’’ were identified with at least two razor/unique peptides. The other 12 proteins were identified with one unique peptide (Table 1).

Among six pembrolizumab samples, the number of host cell proteins and the peak intensities were found higher in the samples purified with gravity flow (sample 1–3) as compared to the samples purified with pressure system (sample 4–6). The highest number of plant proteins were detected in pembrolizumab sample 1 with sixteen proteins identified. Pembrolizumab sample 2 and 3 were detected with three and five plant proteins, respectively. In contrast, two host plant proteins were identified in the sample purified with automated system. It was luminal-binding protein 5 in pembrolizumab sample 4 and cysteine-rich venom protein in pembrolizumab sample 5. Both proteins were detected with low signal intensity. No plant protein was detected in pembrolizumab sample 6. Based on the lower number and signal intensity of host plant proteins detected, this result implies that FPLC pressure system would provide better purification efficiency in terms of host plant protein removal for antibody production as compared to the gravity flow chromatographic system.

In terms of protein intensity, luminal-binding protein 5, RuBisCO large subunit and plastocyanin A’/A’’ were the most abundant host plant proteins detected in pembrolizumab samples. Luminal-binding protein 5 was detected in all three samples purified with gravity flow column and one sample purified with a pressure flow system. RuBisCO large subunit was observed in all three gravity-flow samples but not found in any sample purified with pressure-flow system. Plastocyanin A’/A’’ was found with relatively high intensity in pembrolizumab sample 1. Plant proteins, including luminal-binding protein 5-like, RubisCO small subunit and plastocyanin, were also found in pembrolizumab sample 1, indicating low protein purity of this sample. This result also suggests that plant luminal-binding protein 5, RuBisCO and plastocyanin would be common contaminants in the use of protein A column chromatography. Close examination is further required to monitor those proteins throughout the processes of monoclonal antibody production. Cysteine-rich venom protein, detected with four razor/unique peptides in pembrolizumab sample 5, might be another host plant protein of concern.

**Table 1:** Proteins identified with LC-MS analysis across six pembrolizumab drug products. S1-S3 pembrolizumab products were purified with gravity flow column, and S4-S6 products were purified with FPLC system.

No.	Proteins			Number of razor + unique peptides	% sequence coverage	Intensity					
	Protein ID	Protein name	Species			S1	S2	S3	S4	S5	S6
1	DB09037	Pembrolizumab heavy chain	-	60	89.2	33,627,000	50,735,000	35,365,000	40,549,000	27,951,000	26,532,000
2	DB09037	Pembrolizumab light chain	-	34	100.0	28,631,000	40,199,000	34,344,000	30,315,000	25,128,000	22,130,000
3	Q03685	Luminal-binding protein 5	*N. tabacum*	9	14.6	139,250	368,620	165,300	10,564	0	0
4	P48709	Ribulose biphosphate carboxylase large chain	*N. debneyi*	8	22.5	25,423	22,980	120,500	0	0	0
5	A0A9Y1LRP6	Cysteine-rich venom protein	*N. benthamiana*	4	29.1	0	0	0	0	43,246	0
6	A0A1S4DHM0	Luminal-binding protein 5-like	*N. tabacum*	2	7.4	0	16,237	0	0	0	0
7	P26573	Ribulose bisphosphate carboxylase small subunit, chloroplastic	*N. plumbaginifolia*	2	18.3	24,262	0	10,655	0	0	0
8	P35476	Plastocyanin A’/A’’	*N. tabacum*	2	30.3	139,910	0	0	0	0	0
9	A0A1U7XY18	20 kDa chaperonin, chloroplastic	*N. sylvestris*	1	7.5	17,416	0	0	0	0	0
10	Q42961	Phosphoglycerate kinase, chloroplastic	*N. tabacum*	1	5.0	11,667	0	0	0	0	0
11	A0A1U7W025	20 kDa chaperonin, chloroplastic-like	*N. sylvestris*	1	7.5	13,225	0	0	0	0	0
12	C5J0G6	Phosphopyruvate hydratase	*N. tabacum*	1	6.1	14,618	0	0	0	0	0
13	A0A1S3Z1F3	Glutamate synthase (ferredoxin)	*N. tabacum*	1	2.9	8,829	0	0	0	0	0
14	A0A1S4BNP6	Chaperonin 60 subunit beta 2, chloroplastic	*N. tabacum*	1	3.7	8,857	0	0	0	0	0
15	A0A1U7W8V2	Stromal 70 kDa heat shock-related protein, chloroplastic	*N. sylvestris*	1	4.2	24,381	0	0	0	0	0
16	A0A1S4A1K3	Plastocyanin	*N. tabacum*	1	24.6	72,615	0	0	0	0	0
17	B8R519	Ubiquitin	*N. benthamiana*	1	25.4	11,788	0	9,657	0	0	0
18	P18212	Oxygen-evolving enhancer protein 2–2, chloroplastic	*N. tabacum*	1	10.2	24,810	0	0	0	0	0
19	A0A1U7VQG2	Late blight resistance protein homolog R1A-3 isoform X1	*N. sylvestris*	1	1.2	30,132	0	28,167	0	0	0
20	P27082	Superoxide dismutase [Cu-Zn]	*N. plumbaginifolia*	1	23.7	39,053	0	0	0	0	0

### Peptide details in the identification of recombinant pembrolizumab and *N. benthamiana* host plant proteins

The details of peptides used for identifying pembrolizumab and *N. benthamiana* host cell proteins are presented in Table 2 and Supplementary Table S1. Among 60 and 34 peptides of pembrolizumab heavy and light chains, the peptides with highest MaxQuant score were DVQLLEQVQLVQSGVEVK and VDNALQSGNSQESVTEQDSK, with a score of 361.90 and 328.73, respectively. The peptides with highest score among all host cell proteins were DLAQEGNQIIR and NEGRDLAQEGNQIIR with a score of 142.94 and 139.64, respectively. Both are unique peptides for RuBisCO large subunit protein. The subsequent peptides in the rank were VAAFAQNYANQR (136.39) and FDLTGIAPAPR (132.38), characteristic for luminal-binding protein 5 and cysteine-rich venom protein, respectively. Detection of these peptides with relatively high identification score increased confidence in protein identification. In contrast, low identification score was ascribed for the proteins identified with only one unique and razor peptide and low signal intensity, for example phosphoglycerate kinase (29.92), phosphopyruvate hydratase (32.24) and chaperonin 60 subunit beta 2 (35.90). These proteins were identified with low confidence.

**Fig. 3:**
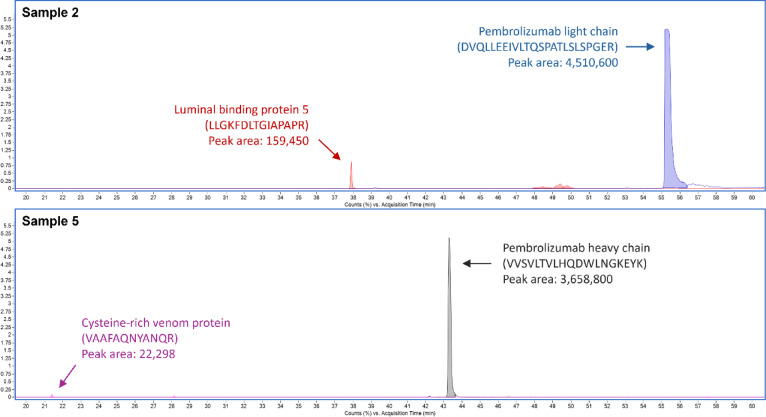
Chromatograms comparing the highest-intensity peptides of recombinant pembrolizumab and *N. benthamiana* host plant proteins. The chromatograms display representatives of protein samples 2 and 5 for the pembrolizumab products purified with manual gravity flow and FPLC system, respectively. The chromatogram of sample 2 compares the peaks between pembrolizumab light chain and plant luminal-binding protein 5. The chromatogram of sample 5 compares the peaks between pembrolizumab heavy chain and cysteine-rich venom protein.

In terms of protein intensity, peptides with the highest peak intensity among pembrolizumab heavy and light chains were VVSVLTVLHQDWLNGKEYK (17,949,000) and DVQLLEEIVLTQSPATLSLSPGER (22,451,000), respectively. Peptides with the highest peak intensity among host plant proteins were LLGKFDLTGIAPAPR (326,520) and LSQEEIER (146,820). These peptides belonged to luminal-binding protein 5. The intensity ratio between the most intense peptides of the host cell protein and the pembrolizumab was highest in sample 2 (0.03535), indicating that host plant protein contamination in this sample could be around 3.5% of pembrolizumab protein (Fig. 3). This ratio indicated an estimated purity of the products purified with gravity flow system. In contrast, the products purified with an automated flow system showed higher protein purity. The highest ratio between the peptides with maximum intensity of pembrolizumab and host cell protein was 0.00609, detected in pembrolizumab sample 5. It was the ratio comparing between the peptides of cysteine-rich venom protein and pembrolizumab heavy chain (Fig. 3 and Supplementary Table S1).

**Table 2:** Peptide details for identifying pembrolizumab and host plant proteins.

Protein ID	Protein name	Peptide sequence	Mass (Da)	Charge	Score	Retention time (min)	Total intensity
DB09037	Pembrolizumab heavy chain	DVQLLEQVQLVQSGVEVK (top score)	2010.100	2;3;4	361.90	43.8	15,194,000
		TTPPVLDSDGSFFLYSRLTVDKSR (lowest score)	2700.376	4	21.385	42.4	41,071
		VVSVLTVLHQDWLNGKEYK (top intensity)	2227.200	2;3;4	179.20	43.4	17,949,000
		KPGASVKVSCK (lowest intensity)	1159.638	3	32.518	10.0	9,817
DB09037	Pembrolizumab light chain	VDNALQSGNSQESVTEQDSK (top score)	2134.961	2;3	328.73	18.0	1,221,600
		EAKVQWKVDNALQSGNSQESVTEQDSK (lowest score)	3004.437	4	15.889	25.8	19,055
		DVQLLEEIVLTQSPATLSLSPGER (top intensity)	2594.38	2;3;4	266.17	55.3	22,451,000
		GVSTSGYSYLHWYQQK (lowest intensity)	1902.890	3	52.855	31.4	12,820
Q03685	Luminal-binding protein 5	FDLTGIAPAPR	1156.624	2	132.38	34.3	11,115
		LSQEEIER	1002.498	2	129.77	15.8	146,820
		NSLETYVYNMR	1388.640	2;3	128.58	31.6	39,204
		DILLLDVAPLTLGIETVGGVMTK	2367.334	2;3	126.18	61.3	95,293
		LLGKFDLTGIAPAPR	1567.909	2;3	107.64	37.9	326,520
		NTVIPTKK	899.544	2	92.653	12.9	22,756
		ITITNDKGR	1016.562	3	46.022	12.7	15,045
		IINEPTAAAIAYGLDKK	1786.983	3	43.642	33.5	19,354
		EAEEFAEEDKKVK	1550.746	3	26.995	14.9	7,622
P48709	Ribulose biphosphate carboxylase large chain	DLAQEGNQIIR	1255.652	2	142.94	25.8	25,847
		NEGRDLAQEGNQIIR	1711.860	3	139.64	23.2	55,086
		DTDILAAFR	1020.524	2	131.27	37.2	6,942
		ALRLEDLR	984.5716	3	67.838	25.1	9,294
		YGRPLLGCTIKPK	1501.844	3;4	56.910	22.8	19,942
		LEGERDITLGFVDLLRDDFVEQDR	2849.420	4	53.030	52.8	16,211
		TFQGPPHGIQVER	1464.747	3	44.245	23.7	25,521
		ELGVPIVMHDYLTGGFTANTSLAHYCR	3021.448	4	26.571	45.3	10,063
A0A9Y1LRP6	Cysteine-rich venom protein	VAAFAQNYANQR	1351.663	2;3	136.39	21.4	22,298
		YGENLAAAFPQLNAAGAVK	1903.979	3	73.735	40.2	12,248
		VCGHYTQVVWR	1403.677	3	49.159	23.8	0
		DYLNAHNAAR	1143.542	3	46.022	14.9	8,700
A0A1S4DHM0	Luminal-binding protein 5-like	LSQEEIDR	988.4825	2	80.676	15.0	7,320
		DLLLLDVTPLSLGIETVGGVMTK	2383.328	3	36.854	61.0	8,917
P26573	Ribulose bisphosphate carboxylase small subunit, chloroplastic	IIGFDNVR	932.508	2	74.929	28.3	24,262
		KYETLSYLPDLSQEQLLSEIEYLLK	3014.574	3	35.170	59.6	14,618
P35476	Plastocyanin A’/A’’	IEVLLGSDDGGLAFVPGNFSVSAGEKITFK	3066.591	3	53.645	51.3	108,860
		IEVLLGSDDGGLAFVPGNFSVSAGEK	2577.296	3	32.904	49.9	31,051
A0A1U7XY18	20 kDa chaperonin, chloroplastic	VAEAEEKTAGGLLLTEAAK	1900.015	3	58.934	29.8	17,416
Q42961	Phosphoglycerate kinase, chloroplastic	VGVASVMSHISTGGGASLELLEGK	2298.189	3	29.918	42.8	11,667
A0A1U7W025	20 kDa chaperonin, chloroplastic-like	VAEVEEKTAGGLFLSEAAK	1948.015	3	85.456	31.7	13,225
C5J0G6	Phosphopyruvate hydratase	AAVPSGASTGIYEALELRDGGSEYLGK	2710.345	3	32.243	39.3	14,618
A0A1S3Z1F3	Glutamate synthase (ferredoxin)	ASDSANLDSAAELLIR	1644.832	2	52.712	39.5	8,829
A0A1S4BNP6	Chaperonin 60 subunit beta 2, chloroplastic	LSGGVAVIQVGAQTETELKEKK	2284.264	4	35.904	31.3	8,857
A0A1U7W8V2	Stromal 70 kDa heat shock-related protein, chloroplastic	SEVFSTAADGQTSVEINVLQGEREFVRDNK	3324.622	4	38.037	38.4	24,381
A0A1S4A1K3	Plastocyanin	ISMSEEDLLNAPGETYSVTLSEKGTYSFYCSPHQGAGMVGK	4440.024	4	27.554	41.8	72,615
B8R519	Ubiquitin	KTITLEVESSDTIDNVK	1890.979	3	96.141	29.0	21,444
P18212	Oxygen-evolving enhancer protein 2–2, chloroplastic	FEDNFDATSNVIVAITPTDKK	2324.154	3	97.732	38.6	24,810
A0A1U7VQG2	Late blight resistance protein homolog R1A-3 isoform X1	LLENIFNQVTTSALK	1689.930	2	43.477	35.5	58,299
P27082	Superoxide dismutase [Cu-Zn]	HAGDLGNITVGEDGTASFTLTDKQIPLAGPQSIIGR	3648.875	4	94.233	44.7	39,053

## Discussion

Host cell proteins are concerned impurities in biopharmaceutical products, along with host cell DNA, RNA, lipids and protein aggregate^19^ because they can induce immunogenicity and cause immune dysregulation in the patients^20,21^. In drug product, host cell protein contamination may be varied due to several factors, such as host organisms, culture conditions, manufacturing process and targeted recombinant proteins produced^22^. One of the factors is that host cell proteins can interact with recombinant proteins via chemical interaction^21^. As previously reported, host cell proteins from Chinese hamster ovarian cells are mostly secreted proteins because cell culture fluid is collected for recombinant protein extraction^22^. In plant molecular farming production, host cell proteins are a mix of secreted and intracellular proteins as whole plant cells are collected from protein extraction^23^. Protein purification step is undertaken to isolate target recombinant protein from host cell proteins. However, insufficient and improper purification steps may not completely eliminate host cell proteins, leading to product contamination^8^. Therefore, determination of host cell protein impurities with highly sensitive and reliable techniques is an important step for the quality control of recombinant protein production.

Based on U.S.P. guideline, immunoassay using polyclonal antibodies is a recommended method for host cell protein determination^8^. However, polyclonal antibodies may not capture all host cell proteins, which could be varied among different production batches. A combination of immunoassay with other techniques, such as LC-MS, is suggested to improve host cell protein detection^19^. This study aimed to study the potential of LC-MS tool to characterize plant protein impurities in plant-produced pembrolizumab antibody, which its application for host cell protein detection in plant molecular farming has not been well explored.

This study also investigated two different types of protein A column chromatography for pembrolizumab purification. As a result, host plant proteins were detected lower in pembrolizumab products purified with FPLC system as compared to the products purified with manual gravity flow. Only two host cell proteins, luminal-binding protein 5 and cysteine-rich venom protein, were detected across three different samples purified with FPLC system. In contrast, 17 host cell proteins were found across three samples, purified with manual gravity flow. In addition, the total signal intensity of host cell proteins found in FPLC-purified samples was lower than that in the samples purified with gravity flow. Based on this lower number and signal intensity of host cell proteins detected in this study, it can be implied that purification system with pressure flow and prepacked column would be more efficient than the gravity flow system coupled with manually packed column to eliminate host plant proteins. Nonetheless, one step of purification using protein A affinity chromatography would not be sufficient to obtain pure pembrolizumab monoclonal antibody. Second chromatography using size exclusion or ion exchange column would be required to improve host cell protein clearance^5^.

In this study, pembrolizumab sample was diluted to 1 mg ml^−1^ for LC-MS analysis. Commercially, the pembrolizumab drug or Keytruda^®^ is formulated at 25 mg ml^−1^ in concentration. The number of host cell proteins detected would be increased if the higher concentration of pembrolizumab sample was analyzed. Based on current data, the ratio between the highest peaks of pembrolizumab against host cell protein was 0.0354 and 0.0061 for the samples purified with gravity flow and pressure flow, respectively. By estimation, the abundance of host cell protein in 1 mg ml^−1^ pembrolizumab samples was approximately 35.4 and 6.1 µg ml^−1^ in the samples purified with gravity flow and pressure flow, respectively. These were much higher than the specification, mentioned in the U.S.P. guideline, that the presence of host cell proteins in recombinant proteins should be less than 100 ng ml^−1^ ^[8]^.

Surprisingly, luminal-binding protein 5 was the most abundant *N. benthamiana* protein observed in this study. It was detected in the proteins purified with both gravity and pressure flows. Generally, luminal-binding protein 5 is highly expressed in the endoplasmic reticulum (ER) lumen. It belongs to the heat shock protein 70 (Hsp70) family with various functions to activate protein folding and untangle protein misfolding^24^. It plays an important role in maintaining ER properties^25^. Increased level of Hsp70 proteins is a key marker for ER stress^26^. In soybean and tobacco, luminal-binding protein 5 was reported to play a role in plant resistance toward drought stress^27^. Interestingly, this protein is known as immunoglobulin-binding protein (BiP), which, in some other organisms, is identified as heat shock 70 kDa protein 5 (HSPA5) and glucose-regulated protein 78 (GRP78)^24^. Human BiP binds with free immunoglobulin heavy chain at the first constant region (C_H_1)^28^. Human BiP has similar functions to plant luminal-binding protein 5 in controlling protein folding and ER functions. The alignment between tobacco luminal-binding protein 5 and human BiP showed 69.68% sequence similarity (Supplementary Data S1). While the alignment between tobacco luminal-binding protein 5 and *A. thaliana* Hsp70 BIP1, BIP2 and BIP3 was approximately 78.50%, implying *Nicotiana* luminal-binding protein 5 is identical to the BiP of other species. According to this information, it could be concluded that the presence of luminal-binding protein 5 in pembrolizumab products upon protein A purification method might be due to the binding of luminal-binding protein 5 to pembrolizumab protein and co-purification with the antibody.

Apart from luminal-binding protein 5, cysteine-rich venom protein was also detected in pembrolizumab sample purified with FPLC system. Its sequence was 95.0% similar to basic form pathogenesis-related protein 1-like (Supplementary Data S1), which is a secretory plant defense protein, involving in plant protection against pathogens^29^. Its presence in purified pembrolizumab was unclear and would require further study to understand the mechanisms of co-purification of cysteine-rich venom protein with pembrolizumab antibody.

RuBisCO is the most abundant protein in plant cells and tentatively the most abundant protein on earth^30^. It is a key enzyme for carbon fixation in photosynthetic activity^31^. It is found in plants, algae, and certain types of protists and bacteria^32^. RuBisCO has not been reported to interact with human immunoglobulin or protein A. The detection of RuBisCO large and small chains in pembrolizumab samples could be an incomplete protein clearance during purification process. RuBisCO was found only in the products purified with gravity flow but not observed in the products purified with FPLC system. The result implies that host plant proteins from *N. benthamiana* can be removed from targeted pembrolizumab monoclonal antibody with protein A column chromatography to a certain degree. The clearance was slightly more effective with the pressure flow system as the lower number and signal intensity of plant proteins were detected in the samples purified with FPLC as compared to the samples purified with gravity flow column. In addition, the detection of host plant proteins in this study has promoted understanding in the mechanisms of plant protein contamination upon recombinant antibody production. This information benefits the selection of secondary chromatography or other purification techniques to obtain high purity pembrolizumab protein.

## Conclusion

This study provided insight into the *N. benthamiana* host plant proteins that could contaminate upon the production of recombinant antibodies within plant molecular farming system. Luminal-binding protein 5, RuBisCO and cysteine-rich venom protein were major host plant proteins detected in the pembrolizumab products. The findings provided better understanding in the mechanisms of host plant protein contamination and could facilitate control of plant protein contamination in the recombinant pembrolizumab production. It also highlighted that close monitoring and accurate examination are important steps for the quality control of recombinant protein production. In addition, it suggested that additional chromatography and/or other purification steps are required to improve protein purification efficiency to yield high-purity monoclonal antibody products. This study also confirmed that LC-MS can be a powerful tool to detect host cell proteins in recombinant protein production as demonstrated in this plant molecular farming system of this study.

## Materials and methods

### Chemicals

Phosphate buffer saline (PBS) contained 0.14 M NaCl, 0.003 M KCl, 0.01 M Na_2_HPO_4_ and 0.002 M KH_2_PO_4_ in a final concentration. Its pH was adjusted to 7.4 with HCl. A 0.01 M glycine buffer, pH 2.9 was prepared by dissolving glycine in water and adjusting pH to 2.9 using HCl. A 0.05 M citrate buffer, pH 3.5 contained 0.087 M tri-sodium citrate dihydrate and 0.013 M citric acid with pH adjustment using HCl. A 50 mM ammonium bicarbonate (ABC) buffer was prepared by diluting ammonium bicarbonate in water without pH adjustment. LC-MS grade water was acquired from Merck, US, and LC-MS grade acetonitrile was acquired from Fisher Scientific, US.

### Plant materials

Seeds of *Nicotiana benthamiana* plant were supported by Dr. Supaart Sirikantaramas, Faculty of Science, Chulalongkorn University. Plants were grown at Baiya Phytopharm Co., Ltd. under controlled environment, where the growth facility was controlled and managed under good manufacturing practice (GMP).

### Cloning of pembrolizumab gene

The sequence of plant-produced pembrolizumab was obtained from DrugBank database (accession no. DB09037) with an addition of DVQLLE sequences at the N-terminus of both heavy chain and light chain to furnish restriction sites for molecular cloning steps. SEKDEL sequence was not added to the C-terminus of pembrolizumab sequence. Therefore, typical plant glycosylation was expected to be a post-translational modification at N-303 glycosylated site. The cloning procedure of pembrolizumab was deliberately described in previous studies^17,33^. Briefly, the nucleotide construct was inserted into pBAIYA geminiviral vector and transformed into *Agrobacterium tumefaciens* using electroporation.

### Recombinant pembrolizumab production

In this study, *Nicotiana benthamiana* transient expression system was applied for producing recombinant pembrolizumab. Bacteria transformation and agroinfiltration method were detailed in previous works^34,35^. Briefly, *N. benthamiana* plants were grown in hydroponic system at 25 ± 2 °C under 16 h light and 8 h darkness with control humidity at 55% RH. Light emitting diode (LED) was a light source. Electrical conductivity was 3,000 ± 1,000 µS cm^−1^. Bacterial *A. tumefaciens*, carrying pBaiya vectors inserted with pembrolizumab sequence of heavy and light chain, were grown at 28 °C for two days with shaking condition. The pBAIYA viral vector was identical to the pBY2eK of the previous works. The pathogen was infiltrated into three-week-old hydroponically grown *N. benthamiana* plants using a vacuum chamber. Infected plants were maintained at 25 °C with 16 h light and 8 h darkness for three days, when wilting symptoms were observed. Plant leaves were collected and extracted using phosphate buffer saline (PBS), followed by high-speed centrifugation at 26,000 ×g for 40 min and filtration through 0.45 μm membrane filter. The ratio of plant weight per extraction buffer was approximately 1 g per 1 ml. Plant extracts were split into six samples and subjected to purification steps separately. They were purified with protein A affinity column chromatography with two different systems, gravity-flow column (three replicates) and prepacked column with pressure-flow system (three replicates). The overall workflow of this study is shown in Fig. 1.

### Protein purification using protein A gravity flow column

Protein A resin, mabSelect prismA (Cytiva, Sweden), was manually packed in a plastic column, 2.0 cm in diameter and 8.5 cm in height. Approximately 3 ml of protein A suspension was loaded into a column with 2-cm height. The column was primarily equilibrated with approximately 10 ml of 1× PBS and loaded with approximately 20 ml of crude protein extract. The column was washed with approximately 30 ml of 1× PBS and finally eluted using approximately 10 ml of 0.1 M glycine buffer, pH 2.9. Protein eluate was neutralized with 1 M Tris-HCl, pH 8.8 and then dialyzed against 1× PBS (cell grade) for three cycles. The dialyzed protein was concentrated using Amicon Ultra-4 30 kDa molecular weight cutoff (MWCO) centrifugal device to a final concentration of 1 mg ml^−1^. Three independent batches of pembrolizumab production were performed for data comparison.

### Protein purification using protein A pressure-flow system

AKTA pure-25 machine (Cytiva, Sweden) equipped with a mabSelect prismA pre-packed column (Cytiva, Sweden) was used for automatic fast protein liquid chromatography (FPLC) purification system. The column was packed with protein A suspension for around 10-cm height. The column was equilibrated with 5 column volumes (CV) of 1× PBS at a flow rate of 1 ml min^−1^. Approximately 50 ml of protein extract (50 g crude plant) was gradually loaded to the column at a flow rate of 1 ml min^−1^, followed by washing with 5 CV of 1× PBS. Finally, target protein was eluted with 5 CV of 0.05 M citrate buffer, pH 3.5. Protein peak was detected with UV 280 nm along the purification. Protein eluate was neutralized with 1.5 M Tris-HCl, pH 8.8 immediately after elution and dialyzed against 1× PBS (cell grade) for three cycles. Finally, it was concentrated using Amicon Ultra-4 30 kDa MWCO device to a final concentration of 1 mg ml^−1^. Likewise, the process was performed in triplicate independently. Although different elution buffers were used for gravity-flow and pressure-flow systems, both were acidic buffer with pH < 3.5 and commonly used for antibody purification with protein A column^36^.

### SDS-PAGE and Western blot

Protein concentration was measured using general Bradford assay. Approximately 3 µg protein were loaded into 4–15% gradient SDS-PAGE gel under reducing and non-reducing conditions. Gel was run at 100 V for approximately 60 min and stained with Coomassie blue. After destaining, gels were imaged using ImageQuant LAS 500 system (GE Healthcare, UK). For Western blot analysis, separate gels were run under similar settings without color staining. They were transferred to nitrocellulose membranes using Mini-Trans-Blot system (Bio-Rad, US). The membrane was blocked with 5% skim milk for 30 min and probed with goat anti-human gamma heavy chain conjugated with horseradish peroxidase (HRP) (Southern Biotech, US) at 1:10,000 dilution or rabbit anti-human kappa light chain conjugated with HRP (Southern Biotech, US) at 1:5,000 dilution for 2 h. The membrane was washed with 1x PBS and exposed to SuperSignal West Pico enhanced chemiluminescent substrate (Thermo Fisher Scientific, US). The developed signal was detected using ImageQuant LAS 500 system (GE Healthcare, UK).

### Protein preparation for LC-MS analysis

Approximately 20 µg of plant-produced pembrolizumab was diluted two times with 50 mM ammonium bicarbonate (ABC) buffer. The protein solution was reduced with 10 mM dithiothreitol (DTT) at 65 °C for 30 min and alkylated with 25 mM iodoacetamide (IAA) at room temperature for 20 min under darkness. Proteins were digested with 0.25 µg trypsin at 37 °C for 4 h. The reaction was stopped using 10% formic acid (FA) and centrifuged at 14,000 rpm for 10 min. Supernatant was transferred to a polypropylene vial and subjected to LC-MS analysis.

### LC-MS analysis

Peptide samples were analyzed using Agilent 1290 Infinity II LC system coupled with Agilent 6545XT Q-TOF mass spectrometer (Agilent Technologies, US). LC separation was conducted on AdvanceBio Peptide Mapping column (120 Å, 2.1 × 150 mm, 2.7 μm, Agilent Technologies, US) at 60 °C. Injection volume was 10 µl. Mobile phase A was 0.1% FA in water and mobile phase B was 0.1% FA in acetonitrile. LC gradient was set as follows; 0% B for 2 min, 0–20% B in 33 min, 20–30% B in 20 min, 30–50% B in 10 min, 50–90% B in 5 min, 90% B for 5 min, 90–0% B in 5 min and 0% B for 5 min, with constant flow rate of 0.4 ml min^−1^. MS analysis was conducted in positive mode with a mass range of 100–1700 m/z. MS parameters were set as follows; gas temperature at 325 °C, nebulizer at 35 psi, dying gas at 13 L min^−1^, sheath gas temperature at 350 °C, sheath gas flow at 12 L min^−1^, capillary voltage at 4000 V, nozzle voltage at 500 V, fragmentor voltage at 175 V and skimmer voltage at 65 V. Acquisition time was 1 spectrum per s. Maximum 10 precursor ions per cycle were selected for MS/MS fragmentation. Collision energy (CE) varied according to the charge state of the peptide. For peptides with charge + 1 and + 2, the CE was calculated using a formula of (3.1 × ((m/z)/100) + 1), while peptides with charge ≥ + 3, the CE was calculated using a formula of (3.6 × ((m/z)/100) − 4.8). Data was collected in centroid mode.

#### Data analysis

Raw files were converted as described in previous study^37^. Firstly, the Agilent.d files were converted to.mzML file using msConvert command in ProteoWizard software (https://proteowizard.sourceforge.io/) with default settings. Then, the.mzML file was converted to.mzxML file using TOPPAS function in OpenMS software (https://openms.de/). Default setting was also applied, where “force MaxQuant compatibility” function was enabled.

Data files in.mzxML format were loaded into MaxQuant software version 2.4. All parameters for Agilent QTOF instrument were applied as default. Trypsin was selected as a digestive enzyme, and two missed cleavage was allowed. Methionine oxidation and N-terminal acetylation were set as a variable modification, while cysteine carbamidomethylation was a fixed modification. Protein database was acquired from UniProt website (https://www.uniprot.org/). All 154,673 *Nicotiana* proteins (taxonomy ID: 4085) were selected. Pembrolizumab heavy and light chain sequences were added to the database. Proteins identified by site and as potential contaminants were removed from the list. Chromatogram was plotted using Agilent MassHunter Qualitative Analysis 10.0 software (Agilent Technologies, US).

### Authorship contributions

R.D. and P.S. conceptualized the study, designed experiments, conducted LC-MS acquisition and data analysis. K.R. performed pembrolizumab cloning and transformation. K.R. performed small-scale purification and provided protein samples. S.V. performed pilot-scale purification and provided protein samples. T.S. performed SDS-PAGE and Western blot analyses. R.D. and P.S. drafted the manuscript. C.J.I.B. formatted the manuscript. W.P. acquired funding and provided supervision. R.D., P.S., C.J.I.B. and W.P. edited the manuscript. All authors approved the manuscript.

## Electronic supplementary material

Below is the link to the electronic supplementary material.

Supplementary Material 1

Supplementary Material 2

Supplementary Material 3

## Data Availability

Data of this study is included in this article and supplementary information. Further inquiries can be made to the corresponding authors.
