# Supplementary figures and images for: LC-MS determination of Nicotiana benthamiana host plant proteins in the drug products of recombinant plant-produced pembrolizumab
Source: Sci Rep. 2025 Jul 15;15:25635. doi: 10.1038/s41598-025-11541-6 (PMC12264258; doi:10.1038/s41598-025-11541-6)

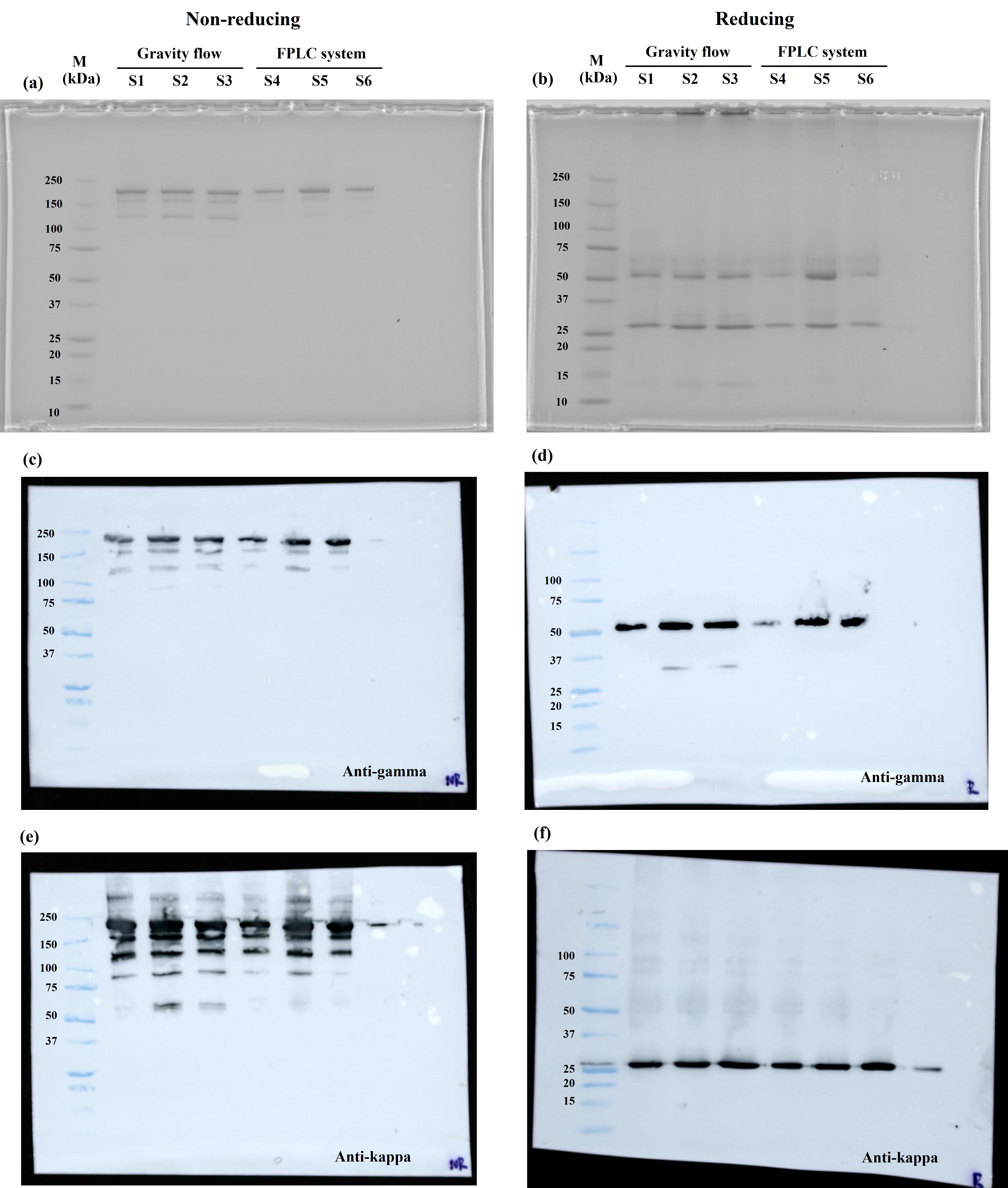

Supplement: Supplementary file 1 — Supplementary Material 1 [file 41598_2025_11541_MOESM1_ESM.jpg]
