# Supplementary material for: LC-MS determination of Nicotiana benthamiana host plant proteins in the drug products of recombinant plant-produced pembrolizumab
Source: Sci Rep. 2025 Jul 15;15:25635. doi: 10.1038/s41598-025-11541-6 (PMC12264258; doi:10.1038/s41598-025-11541-6)
Supplement: Supplementary file 2 — Supplementary Material 2 [file 41598_2025_11541_MOESM2_ESM.docx]

**Supplementary Data S1.** Alignments of *Nicotiana* luminal-binding protein 5 and cysteine-rich venom protein to the related proteins of other species.

**Alignment between tobacco luminal-binding protein 5 (Q03685) and human immunoglobulin-binding protein (BiP; P11021), showing 69.68% similarity**

BIP5_TOBAC MAGAWKRRASLIVFAIVLFGSLFAFSIAKEEATKLGTVIGIDLGTTYSCVGVYKNGHVEI 60

BIP_HUMAN ------MKLSLVAAMLLLLS--AARAEEEDKKEDVGTVVGIDLGTTYSCVGVFKNGRVEI 52

: **:. ::*:. * : ::: .:***:*************:***:***

BIP5_TOBAC IANDQGNRITPSWVAFT-DGERLIGEAAKNQAAVNPERTIFDVKRLIGRKFDDKEVQRDK 119

BIP_HUMAN IANDQGNRITPSYVAFTPEGERLIGDAAKNQLTSNPENTVFDAKRLIGRTWNDPSVQQDI 112

************:**** :******:***** : ***.*:**.******.::* .**:*

BIP5_TOBAC KLVPYEIVNKDGKPYIQVKIKDGETKVFSPEEISAMILTKMKETAEAYLGKKIKDAVVTV 179

BIP_HUMAN KFLPFKVVEKKTKPYIQVDIGGGQTKTFAPEEISAMVLTKMKETAEAYLGKKVTHAVVTV 172

*::*:::*:*. ******.* .*:**.*:*******:***************:..*****

BIP5_TOBAC PAYFNDAQRQATKDAGVIAGLNVARIINEPTAAAIAYGLDKKGGEKNILVFDLGGGTFDV 239

BIP_HUMAN PAYFNDAQRQATKDAGTIAGLNVMRIINEPTAAAIAYGLDKREGEKNILVFDLGGGTFDV 232

****************.****** *****************: *****************

BIP5_TOBAC SILTIDNGVFEVLATNGDTHLGGEDFDQRIMEYFIKLIKKKHGKDISKDNRALGKLRREA 299

BIP_HUMAN SLLTIDNGVFEVVATNGDTHLGGEDFDQRVMEHFIKLYKKKTGKDVRKDNRAVQKLRREV 292

*:**********:****************:**:**** *** ***: *****: *****.

BIP5_TOBAC ERAKRALSSQHQVRVEIESLFDGVDFSEPLTRARFEELNNDLFRKTMGPVKKAMEDAGLE 359

BIP_HUMAN EKAKRALSSQHQARIEIESFYEGEDFSETLTRAKFEELNMDLFRSTMKPVQKVLEDSDLK 352

*:**********.*:****:::* **** ****:***** ****.** **:*.:**:.*:

BIP5_TOBAC KNQIDEIVLVGGSTRIPKVQQLLKDYFDGKEPNKGVNPDEAVAYGAAVQGGILSGEGGDE 419

BIP_HUMAN KSDIDEIVLVGGSTRIPKIQQLVKEFFNGKEPSRGINPDEAVAYGAAVQAGVLSGD--QD 410

*.:***************:***:*::*:****.:*:*************.*:***: ::

BIP5_TOBAC TKDILLLDVAPLTLGIETVGGVMTKLIPRNTVIPTKKSQVFTTYQDQQTTVTISVFEGER 479

BIP_HUMAN TGDLVLLDVCPLTLGIETVGGVMTKLIPRNTVVPTKKSQIFSTASDNQPTVTIKVYEGER 470

* *::****.**********************:******:*:* .*:* ****.*:****

BIP5_TOBAC SLTKDCRLLGKFDLTGIAPAPRGTPQIEVTFEVDANGILNVKAEDKASGKSEKITITNDK 539

BIP_HUMAN PLTKDNHLLGTFDLTGIPPAPRGVPQIEVTFEIDVNGILRVTAEDKGTGNKNKITITNDQ 530

**** :***.****** *****.********:*.****.*.****.:*:.:*******:

BIP5_TOBAC GRLSQEEIERMVKEAEEFAEEDKKVKERIDARNSLETYVYNMRNQINDKDKLADKLESDE 599

BIP_HUMAN NRLTPEEIERMVNDAEKFAEEDKKLKERIDTRNELESYAYSLKNQIGDKEKLGGKLSSED 590

.**: *******::**:*******:*****:**.**:*.*.::***.**:**..**.*::

BIP5_TOBAC KEKIETATKEALEWLDDNQSAEKEDYDEKLKEVEAVCNPIITAVYQRSGGAPGGASEESN 659

BIP_HUMAN KETMEKAVEEKIEWLESHQDADIEDFKAKKKELEEIVQPIISKLYGSAGPPPTGEEDTAE 650

**.:*.*.:* :***:.:*.*: **:. * **:* : :***: :* :* * * .: ::

BIP5_TOBAC EDDDSHDEL 668

BIP_HUMAN KDEL----- 654

:*:

**Alignment between tobacco luminal-binding protein 5 (Q03685) and *Arabidopsis thaliana* heat shock 70 kDa protein BIP1 (Q9LKR3), BIP2 (Q39043) and BIP3 (Q8H1B3), showing 78.79%, 78.48% and 78.30% similarity, respectively**

BIP5_TOBAC ----------MAGAWKRRASLIVFAI----VLFGS-LFAFSIAKEEATKLGTVIGIDLGT 45

BIP1_ARATH ----------MARSFG-ANSTVVLAI----IFFGC-LFALSSAIEEATKLGSVIGIDLGT 44

BIP2_ARATH ----------MARSFG-ANSTVVLAI----IFFGC-LFAFSTAKEEATKLGSVIGIDLGT 44

BIP3_ARATH MIFIKENTAKMTRNKA-IACLVFLTVLDFLMNIGAALMSSLAIEGEEQKLGTVIGIDLGT 59

*: . :.::: : :*. *:: * ***:********

BIP5_TOBAC TYSCVGVYKNGHVEIIANDQGNRITPSWVAFTDGERLIGEAAKNQAAVNPERTIFDVKRL 105

BIP1_ARATH TYSCVGVYKNGHVEIIANDQGNRITPSWVGFTDSERLIGEAAKNQAAVNPERTVFDVKRL 104

BIP2_ARATH TYSCVGVYKNGHVEIIANDQGNRITPSWVGFTDSERLIGEAAKNQAAVNPERTVFDVKRL 104

BIP3_ARATH TYSCVGVYHNKHVEIIANDQGNRITPSWVAFTDTERLIGEAAKNQAAKNPERTIFDPKRL 119

********:* ******************.*** ************* *****:** ***

BIP5_TOBAC IGRKFDDKEVQRDKKLVPYEIVNKDGKPYIQVKIKDGETKVFSPEEISAMILTKMKETAE 165

BIP1_ARATH IGRKFEDKEVQKDRKLVPYQIVNKDGKPYIQVKIKDGETKVFSPEEISAMILTKMKETAE 164

BIP2_ARATH IGRKFEDKEVQKDRKLVPYQIVNKDGKPYIQVKIKDGETKVFSPEEISAMILTKMKETAE 164

BIP3_ARATH IGRKFDDPDVQRDIKFLPYKVVNKDGKPYIQVKVK-GEEKLFSPEEISAMILTKMKETAE 178

*****:* :**:* *::**::************:* ** *:*******************

BIP5_TOBAC AYLGKKIKDAVVTVPAYFNDAQRQATKDAGVIAGLNVARIINEPTAAAIAYGLDKKGGEK 225

BIP1_ARATH AYLGKKIKDAVVTVPAYFNDAQRQATKDAGVIAGLNVARIINEPTAAAIAYGLDKKGGEK 224

BIP2_ARATH AYLGKKIKDAVVTVPAYFNDAQRQATKDAGVIAGLNVARIINEPTAAAIAYGLDKKGGEK 224

BIP3_ARATH AFLGKKIKDAVITVPAYFNDAQRQATKDAGAIAGLNVVRIINEPTGAAIAYGLDKKGGES 238

*:*********:******************.******.*******.*************.

BIP5_TOBAC NILVFDLGGGTFDVSILTIDNGVFEVLATNGDTHLGGEDFDQRIMEYFIKLIKKKHGKDI 285

BIP1_ARATH NILVFDLGGGTFDVSVLTIDNGVFEVLSTNGDTHLGGEDFDHRVMEYFIKLIKKKHQKDI 284

BIP2_ARATH NILVFDLGGGTFDVSVLTIDNGVFEVLSTNGDTHLGGEDFDHRIMEYFIKLIKKKHQKDI 284

BIP3_ARATH NILVYDLGGGTFDVSILTIDNGVFEVLSTSGDTHLGGEDFDHRVMDYFIKLVKKKYNKDI 298

****:**********:***********:*.***********:*:*:*****:***: ***

BIP5_TOBAC SKDNRALGKLRREAERAKRALSSQHQVRVEIESLFDGVDFSEPLTRARFEELNNDLFRKT 345

BIP1_ARATH SKDNKALGKLRRECERAKRALSSQHQVRVEIESLFDGVDFSEPLTRARFEELNNDLFRKT 344

BIP2_ARATH SKDNKALGKLRRECERAKRALSSQHQVRVEIESLFDGVDLSEPLTRARFEELNNDLFRKT 344

BIP3_ARATH SKDHKALGKLRRECELAKRSLSNQHQVRVEIESLFDGVDFSEPLTRARFEELNMDLFKKT 358

***::********.* ***:**.****************:************* ***:**

BIP5_TOBAC MGPVKKAMEDAGLEKNQIDEIVLVGGSTRIPKVQQLLKDYFDGKEPNKGVNPDEAVAYGA 405

BIP1_ARATH MGPVKKAMDDAGLQKSQIDEIVLVGGSTRIPKVQQLLKDFFEGKEPNKGVNPDEAVAYGA 404

BIP2_ARATH MGPVKKAMDDAGLQKSQIDEIVLVGGSTRIPKVQQLLKDFFEGKEPNKGVNPDEAVAYGA 404

BIP3_ARATH MEPVKKALKDAGLKKSDIDEIVLVGGSTRIPKVQQMLKDFFDGKEPSKGTNPDEAVAYGA 418

* *****:.****:*.:******************:***:*:****.**.**********

BIP5_TOBAC AVQGGILSGEGGDETKDILLLDVAPLTLGIETVGGVMTKLIPRNTVIPTKKSQVFTTYQD 465

BIP1_ARATH AVQGGILSGEGGDETKDILLLDVAPLTLGIETVGGVMTKLIPRNTVIPTKKSQVFTTYQD 464

BIP2_ARATH AVQGGILSGEGGDETKDILLLDVAPLTLGIETVGGVMTKLIPRNTVIPTKKSQVFTTYQD 464

BIP3_ARATH AVQGGVLSGEGGEETQNILLLDVAPLSLGIETVGGVMTNIIPRNTVIPTKKSQVFTTYQD 478

*****:******:**::*********:***********::********************

BIP5_TOBAC QQTTVTISVFEGERSLTKDCRLLGKFDLTGIAPAPRGTPQIEVTFEVDANGILNVKAEDK 525

BIP1_ARATH QQTTVSIQVFEGERSLTKDCRLLGKFDLNGIPPAPRGTPQIEVTFEVDANGILNVKAEDK 524

BIP2_ARATH QQTTVSIQVFEGERSLTKDCRLLGKFDLTGVPPAPRGTPQIEVTFEVDANGILNVKAEDK 524

BIP3_ARATH QQTTVTINVYEGERSMTKDNRELGKFDLTGILPAPRGVPQIEVTFEVDANGILQVKAEDK 538

*****:*.*:*****:*** * ******.*: *****.***************:******

BIP5_TOBAC ASGKSEKITITNDKGRLSQEEIERMVKEAEEFAEEDKKVKERIDARNSLETYVYNMRNQI 585

BIP1_ARATH ASGKSEKITITNEKGRLSQEEIDRMVKEAEEFAEEDKKVKEKIDARNALETYVYNMKNQV 584

BIP2_ARATH ASGKSEKITITNEKGRLSQEEIDRMVKEAEEFAEEDKKVKEKIDARNALETYVYNMKNQV 584

BIP3_ARATH VAKTSQSITITNDKGRLTEEEIEEMIREAEEFAEEDKIMKEKIDARNKLETYVYNMKSTV 598

.: .*:.*****:****::***:.*::********** :**:***** ********:. :

BIP5_TOBAC NDKDKLADKLESDEKEKIETATKEALEWLDDNQSAEKEDYDEKLKEVEAVCNPIITAVYQ 645

BIP1_ARATH NDKDKLADKLEGDEKEKIEAATKEALEWLDENQNSEKEEYDEKLKEVEAVCNPIITAVYQ 644

BIP2_ARATH SDKDKLADKLEGDEKEKIEAATKEALEWLDENQNSEKEEYDEKLKEVEAVCNPIITAVYQ 644

BIP3_ARATH ADKEKLAKKISDEDKEKMEGVLKEALEWLEENVNAEKEDYDEKLKEVELVCDPVIKSVYE 658

**:***.*:..::***:* . *******::* .:***:********* **:*:*.:**:

BIP5_TOBAC RSGGAPGGASEESN--EDDDSHDEL 668

BIP1_ARATH RSGGAPGGAGGESSTEEEDESHDEL 669

BIP2_ARATH RSGGAPG-AGGESSTEEEDESHDEL 668

BIP3_ARATH KTEGENEDD--------DGDDHDEL 675

:: * :.:.****

**Alignment between *Nicotiana benthamiana* cysteine-rich venom protein (A0A9Y1LRP6) and *Nicotiana tabacum* basic form of pathogenesis-related protein 1-like (A0A1S4AL11), showing 94.97% similarity**

tr|A0A9Y1LRP6|A0A9Y1LRP6_NICBE MGYSKTLVACFITFAILSPSSQAQNSPRDYLNAHNAARRQVGVGPMTWDNRVAAFAQNYA 60

tr|A0A1S4AL11|A0A1S4AL11_TOBAC MGYSTTLVACFITFAILFPSSQAQNSPQDYLNAHNAARRRVGVGPMTWDNRVAAFAQNYA 60

****.************ *********:***********:********************

tr|A0A9Y1LRP6|A0A9Y1LRP6_NICBE NQRAGDCRMQHSGGRYGENLAAAFPQLNAAGAVKMWVDEKQFYNYNSNTCAPGKVCGHYT 120

tr|A0A1S4AL11|A0A1S4AL11_TOBAC NQRAGDCRMQHSGGPYGENLAAAFPQLNAAGAVKMWVDEKQFYDYNSNSCAAGKVCGHYT 120

************** ****************************:****:** ********

tr|A0A9Y1LRP6|A0A9Y1LRP6_NICBE QVVWRNSVRLGCARVRCNNGWYFITCNYDPPGNWRGQRPYGDLEEQQPFDSKLELPTDV 179

tr|A0A1S4AL11|A0A1S4AL11_TOBAC QVVWRNSVRLGCARVRCNNGWYFITCNYDPPGNWRGQRPYGDLAEQQPFDSKLELPTDV 179

******************************************* ***************
